# Onward transmission of viruses: how do viruses emerge to cause epidemics after spillover?

**DOI:** 10.1098/rstb.2019.0017

**Published:** 2019-08-12

**Authors:** Brian R. Wasik, Emmie de Wit, Vincent Munster, James O. Lloyd-Smith, Luis Martinez-Sobrido, Colin R. Parrish

**Affiliations:** 1 Baker Institute for Animal Health, Department of Microbiology and Immunology, College of Veterinary Medicine, Cornell University, Ithaca, NY 14853, USA; 2 Laboratory of Virology, Division of Intramural Research, National Institute of Allergy and Infectious Diseases, National Institutes of Health, Hamilton, MT 59840, USA; 3 Department of Ecology and Evolutionary Biology, University of California, Los Angeles, Los Angeles, CA 9095-7239, USA; 4 Fogarty International Center, National Institutes of Health, Bethesda, MD 20892, USA; 5 Department of Microbiology and Immunology, University of Rochester, Rochester, NY 14642, USA

**Keywords:** epidemic emergence, virus, HIV, influenza, parvovirus, host range

## Abstract

The critical step in the emergence of a new epidemic or pandemic viral pathogen occurs after it infects the initial spillover host and then is successfully transmitted onwards, causing an outbreak chain of transmission within that new host population. Crossing these choke points sets a pathogen on the pathway to epidemic emergence. While many viruses spill over to infect new or alternative hosts, only a few accomplish this transition—and the reasons for the success of those pathogens are still unclear. Here, we consider this issue related to the emergence of animal viruses, where factors involved likely include the ability to efficiently infect the new animal host, the demographic features of the initial population that favour onward transmission, the level of shedding and degree of susceptibility of individuals of that population, along with pathogen evolution favouring increased replication and more efficient transmission among the new host individuals. A related form of emergence involves mutations that increased spread or virulence of an already-known virus within its usual host. In all of these cases, emergence may be due to altered viral properties, changes in the size or structure of the host populations, ease of transport, climate change or, in the case of arboviruses, to the expansion of the arthropod vectors. Here, we focus on three examples of viruses that have gained efficient onward transmission after spillover: influenza A viruses that are respiratory transmitted, HIV, a retrovirus, that is mostly blood or mucosal transmitted, and canine parvovirus that is faecal:oral transmitted. We describe our current understanding of the changes in the viruses that allowed them to overcome the barriers that prevented efficient replication and spread in their new hosts. We also briefly outline how we could gain a better understanding of the mechanisms and variability in order to better anticipate these events in the future.

This article is part of the theme issue ‘Dynamic and integrative approaches to understanding pathogen spillover’.

## 1. Emergence of viruses as epidemic pathogens of new hosts

### (a) Viruses in reservoirs

Recent virus discovery projects using various deep sequencing approaches have revealed a large number of distinct viruses and virus-associated sequences in animals of all types, including vertebrates and invertebrates, and there are likely millions of viruses in mammals and birds (e.g. [1–4]). Most animal viruses appear to have defined host ranges, circulating and maintaining themselves among a specific group of animal hosts in established relationships. Spillovers occur when viruses infect hosts in which they are not commonly found, and most often involve the infection of single animal species with limited (or no) transmission to additional animals of the same species [5]. However, the true host ranges (i.e. the animal species that are naturally infected and which can sustain transmission) and onward transmission potentials of many viruses are still poorly defined, because in the past, we did not sample to detect mild or subclinical infections, which appear to be quite common. In addition, our current understanding of the true natural host ranges of most viruses is likely incomplete due to the limited exposure of many animals which are geographically or ecologically isolated from the reservoir species in which the virus naturally circulates.

### (b) Viral infections of animals and the nature of reservoirs

While disease ecologists have debated the best definition of a reservoir, one useful definition is an animal population that can maintain a virus in circulation and which may transmit to a target species such as humans [6,7]. However, it is still notoriously difficult to identify true reservoirs in natural systems, and furthermore, our knowledge of the viruses infecting different animal species and populations has been changing rapidly in recent years, but we have little information about the likelihood of human infection for most viruses of animals. Before the advent of high-throughput sequencing techniques, most viruses were known due to their associations with disease or as a result of isolations in laboratory culture. More recently, unbiased metagenomic analysis of animal-associated samples, including tissues and faeces, has resulted in the discovery of many more viruses based on finding their nucleic acid sequences [1–3]. Most of the viruses now being identified likely have low or no pathogenicity in their natural hosts and are of unknown risk to humans. However, we have clear examples from a number of examples of viruses that cause little disease in their reservoirs but which are highly pathogenic when transferred to new hosts, causing serious disease outbreaks. Among the known emerged viruses in humans, this appears to be true for Ebola virus, Marburg virus, Severe Acute Respiratory Syndrome (SARS) and Middle East Respiratory Syndrome (MERS) coronaviruses, Nipah virus, Hendra virus and avian influenza A viruses (IAV). In addition to emergence in humans, there are many cases of viruses transferring between animals to cause outbreaks or epidemics in new hosts. Some human viruses can also be transferred to animals (as reverse zoonoses) to cause outbreaks directly, or to recombine or re-assort with sequences from other viruses of those animals to introduce altered host range properties—this has been commonly observed for IAVs [8], as well as for metapneumovirus [9]. Some animals can be infected simultaneously by different viruses from humans and other animals, allowing recombination or reassortment, and those animals may therefore sometimes be referred to as ‘mixing vessels’ [10]. Those resulting viruses may have new host range, antigenic or transmission properties compared to either of their parental strains.

### (c) Examples of emerging epidemic viruses

Here, we will outline some of the steps in viral emergence, and then present summaries of three well-documented examples where viruses spilling over from one host to another resulted in a prolonged epidemic or pandemic in the human population, or in dogs. However, there are a number of viruses that are well documented to have emerged to cause pandemics or sustained epidemics (table 1).

**Table 1.** Viruses that are known to have emerged in new hosts and which have caused epidemics or pandemics.

virus	host combinations	review references
Influenza A virus	avian to human, swine, horse, seal, dog. Swine to human, horse to dog	[11]
Human immunodeficiency virus	non-human primate to human, two major events	[12]
Ebola virus	bat to human, outbreaks, some extended epidemics bat to gorilla (to human) bat to duiker (to human)	[13]
MERS coronavirus	camel to human, mostly spillover, some outbreaks	[14]
SARS coronavirus	bat to palm civet to human, global spread but controlled	[14]
Nipah virus	bat to swine epidemic, spillover to humans	[15,16]
Canine parvovirus (CPV)	carnivore to dog pandemic	[17]
Zika virus	primate to human, adaptation to mosquito vector and humans resulted in epidemic	[18]

### (d) Viral cross-host exposures

Humans and other animals are constantly exposed to viruses shed from other animals, yet the vast majority of virus exposures never result in any detectable infection in humans [5]. For example, humans are frequently exposed to viruses of domesticated animals and to those of animals that live in our environment—but most never cause infections. Those exposures may be increasing due to anthopogenic effects (including but not limited to habitat destruction or climate change). Even for many known zoonotic viruses, it is likely that hundreds or even thousands of people are exposed for every person who is infected and who develops detectable disease. It is not always clear where the block to infection occurs, but some of the different possible barriers that viruses may encounter and overcome have been reviewed in [5]. However, it is clear that infection may be blocked at one or more steps in the infection process, including physical barriers, mucus of several types, ciliary and other clearance processes, natural antibodies to some viral glycoproteins or other viral components, innate immune responses that block virus establishment and infection, replication or shedding.

Beyond the initial spillover infection, there is likely a second level of adaptation that is required to spread efficiently and cause an epidemic or pandemic. This review addresses both steps leading to successful emergence by outlining the host and viral factors that were associated with the emergence of three different viruses which have given rise to pandemics in new hosts. Those include an enveloped negative-sense single-stranded RNA virus (IAV), an enveloped RNA-genome retrovirus that replicates through a DNA intermediate (HIV) and a non-enveloped small single-stranded DNA (ssDNA) virus that replicates using host DNA polymerases (canine parvovirus (CPV)). Despite their different structures and replication strategies, each of those emerging viruses had to overcome common barriers to efficiently replicate and transmit in their new hosts, so that they illuminate the common, as well as the virus-unique, processes involved. Because of the limited space and the vast literatures that describe many of these viruses and their properties, we largely reference selected recent reviews; many specific references are contained within those reviews.

## 2. Influenza A virus spillover and epidemic emergence

IAVs are often at the top of most lists of emerging viruses, having jumped into prominence in 1918 with the global pandemic caused by the H1N1 strain in humans and in swine [19,20]. This was followed by the emergence of the pandemic H2N2, H3N2 and H1N1 pandemic strains of IAV in humans in 1957, 1968 and 2009, respectively [11,21]. Other epidemic strains have arisen in horses (H7N7 and H3N8), swine (besides the H1N1, there have been two different H3N2 strains and reassortant forms [22]), seals and dogs (H3N8 and H3N2) [23–25]. Although there are IAVs in bats, those strains are not known to spread to other hosts [26].

Birds in fresh or salt water environments are the primary reservoirs of IAVs where infections are largely non-pathogenic, replicating in the gastrointestinal tract, and the viruses are shed into the water where they are taken up, likely by oral and respiratory routes, to infect other individuals [11,21]. Apart from the bat viruses, all IAVs in mammalian species, including humans, ultimately originate from viruses in wild birds, either directly or via intermediate hosts.

The origins of the 1918 H1N1 virus are still not well documented, but the virus likely arose shortly before 1918 when an avian virus was transmitted to humans or swine. However, we have little information about the viruses circulating in birds or in other hosts prior to 1918, and it is also not clear if the 1918 IAV emerged in humans and then transferred to swine, or vice versa [21]. However, the H2N2 pandemic strain emerged in 1957 when the haemagglutinin (HA), neuraminidase (NA) and PB2 gene segments of the H1N1 virus were replaced by reassortment with one or more avian viruses, while in 1968, the HA and PB2 gene segments of that H2N2 virus were in turn replaced by those from an avian virus, giving rise to the H3N2 strain [11,22,27]. The H1N1 pandemic virus that emerged in 2009 had a complex history and contained segments from a variety of different host sources, including segments with swine, avian and human viral origins, and likely originated in swine in Mexico [10,28].

### (a) Barriers to viral emergence

**Figure 1.**
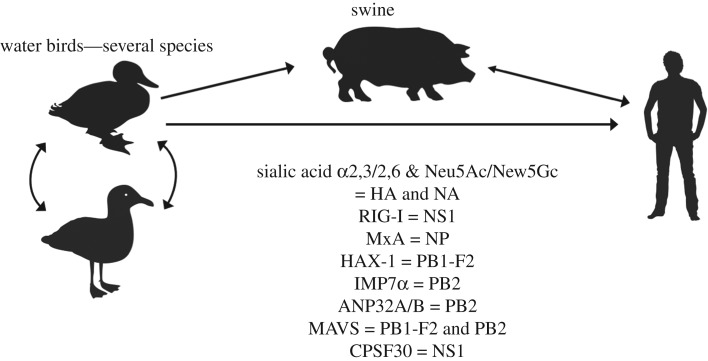
The emergence of pandemic IAV strains in humans, showing known host relationships, host proteins that influence infection and the viral proteins that are affected or which allow a barrier to be overcome—their exact roles and order of contact and evasion are not specifically shown. The possible role of swine as an intermediate host is indicated, but the adaptation process in humans versus swine is still unclear.

While spillovers by IAV are quite common, epidemics are relatively rare, and making the shift from an intestinal infection and faecal–oral transmission in water birds to a respiratory infection and aerosol transmission in humans or other mammals would appear to be a significant problem. Several different host barriers have been identified that were overcome by one or more emerging IAVs, and those have been recently comprehensively reviewed [11], and also an assessment of pandemic risk has been developed [29]. The main pathways to human emergence are outlined in figure 1. Each epidemic in humans and most other mammals has initiated with a single virus genotype, and that virus is assumed to have overcome a number of barriers to become a well-adapted and transmissible virus of mammals. Across these known examples of animal influenza viruses overcoming host-specific differences to cause epidemics in humans, genetic changes have been identified in almost every step of the IAV replication cycle. Those include the animal environments that differ between the avian gastrointestinal tract which is around 41°C, and the upper or lower respiratory tracts of mammals, which are approximately 34 or approximately 37°C. Other differences include the display and binding of specific sialic acid receptors and linkage forms which influence haemagglutinin (HA) binding and neuraminidase (NA) cleavage, release of the viral ribonucleoproteins from the endosome and transport to the nucleus, RNA replication and/or transcription, antagonizing various interferon-associated innate immune responses, viral budding from the cell and release from the cell surface, shedding and transmission among individuals of that host [11].

### (b) Binding to the host receptor

The receptor for IAV is described as sialic acid (Sia), but on more detailed analysis, it is clear that the IAV receptor is complex and may vary in form between hosts [11,30,31]. The Sia found on human cells is the *N*-acetyl neuraminic acid, but in other hosts modified forms such as *N*-glycolyl neuraminic acid are also commonly found, while in some (horses and guinea pigs), there are high levels of an inhibitory Sia, Neu4,5Ac. Those structural differences may influence the binding of HA, and also inhibit the sialidase activity of the viral NA. A second level of receptor specificity comes from the linkage of the Sia to the glycan, which is commonly α2,6 in the upper respiratory tract (and a combination of α2,3 and α2,6 in the lower respiratory tract) in humans, but is most often α2,3 in the intestinal tract of birds and some other hosts. The switch in specificity from the α2,3 to the α2,6 Sia linkage seems to be a requirement for efficient human-to-human transmission [30]. A third receptor-associated inter-host difference is the length and branching of the glycan, and the number of Sia that are displayed [32,33]. This may affect the binding of HA to the receptor as more branched and multi-Sia glycans can bind virus with higher affinity. The length of the glycan may also require a change in the structure of the NA tetramer, which tends to have shorter sequences in the stalk structure in viruses growing in avian tissues, and longer when the longer glycans in the human respiratory tract are encountered [11].

### (c) Nuclear entry, replication and export

Once the virus has successfully bound to the receptors in the new host and entered the cell, it needs to replicate successfully in the new intracellular environment. IAV replicates in the nucleus of the host and its genome thus needs to be imported into the nucleus using the cellular transport machinery. The IAV PB2 and nucleoprotein proteins interact with the nuclear entry protein importin-α in a host-specific manner, and this requires adaption of these viral proteins to the human or other host cells [34].

Optimal viral polymerase activity depends on interaction of viral ribonucleoproteins with host proteins, and a mutation of the PB2 protein at residue 627 from Glu to Lys is commonly observed in viruses adapted to replicate in humans, due at least in part to the necessity to interact with the cellular protein acidic leucine-rich nuclear phosphoprotein 32 (ANP32), which also binds other cellular proteins [11,35]. Nuclear export is a function of the viral nuclear export protein, and this may again require adaption of the virus to the new host [36].

### (d) Antagonizing innate immunity

Once taken into the cell, there are additional host factors that interact with the IAV components in a host-specific manner [11,37]. While several important host factors have been identified, three that are well characterized are the tripartite motif-containing protein 25 (TRIM25) and the retinoic acid-inducible gene I (RIG-I), which can bind the incoming viral ribonuleoproteins and reduce infection, and the MxA protein (in humans) which is a nuclear interferon-stimulated gene (ISG) that binds to the viral ribonuleoproteins to block infection. Viruses from birds or other hosts may be able to evade those factors through the acquisition of specific mutations in PB2 or nucleoprotein. During replication, IAVs induce additional host immune responses, in particular the interferon response, which triggers the expression of many different interferon-stimulated genes that can block many steps of the viral replication cycle. The IAV non-structural 1 protein is a multipurpose protein that can block or downregulate many of those responses, and evolution of NS1 occurs after transfer into new hosts to optimize the antagonism of the host innate immune response [11,37,38]. Some of these changes are likely to be involved in fine tuning the virus:host interaction after pandemic spread has already been accomplished [39]. For example, the ability of IAV NS1 to interact with the cellular cleavage and polyadenylation-specific factor 30 and inhibit host gene expression and interfering with the Janus kinase/signal transducers and activators of transcription (JAK/STAT) pathway have been shown to be an important mechanism of IAV host adaptation [40].

### (e) Particle release and host-to-host transmission of influenza A virus

Many IAVs cause severe infections of new animal hosts, including humans, but without being able to be shed and transmit efficiently. Virion production processes occur with the viral ribonuleoproteins budding from the plasma membrane, and viral release requires the activity of the viral NA, which cleaves the sialic acids on viral glycoproteins and on host materials to allow particles to disaggregate and release efficiently. The processes underlying the transmission of influenza viruses are still not well understood, but likely require optimal receptor interactions and replication, along with the ability to survive in the respiratory secretions of the new host. In humans, the respiratory secretions that the virus is shed in have a relatively low pH (around 5.5), and a number of mutations in the interior of the HA trimer have been found that stabilize the HA at lower pHs and have been associated with the ability to transmit efficiently [41,42].

## 3. HIV strains and their emergence in humans after transfer from primate lentiviruses

HIV is the cause of acquired immune deficiency syndrome (AIDS) in humans, and emerged as a new infection and disease in humans during the twentieth century, becoming widespread around the world in the late 1970s and early 1980s. There are a number of related strains of HIV, with the HIV-1 M strain spreading around the world and caused a pandemic, and the O strain spreading widely in Central Africa, while others (N and P) more likely represent spillover infections with limited transmission [12].

HIV has killed more than 40 million people through the development of AIDS and infections that are facilitated by the viral immunosuppression, and around 1.8 million people are still being infected each year despite the development and use of effective antiviral drugs [43]. HIV is a lentivirus within the retrovirus family, meaning that it packages two copies of the RNA genome, along with a reverse transcriptase that converts the genome into DNA once the virus enters the cell; the HIV DNA genome is then integrated into the host chromosome, and new viral genomic RNA and mRNAs are produced from that integrated copy.

The background to the emergence of two HIV strains as an epidemic diseases in humans are now quite well understood, and many of the barriers and processes involved have been recently reviewed in depth [12,44,45]. These viral emergences clearly illustrate many of the points that are in common with other viral epidemic emergence events.

### (a) Cross-species transmission and genetic recombination

The ancestral viruses that eventually gave rise to HIV were common and well-established simian immunodeficiency viruses (SIV) in old world monkeys and hominoids, which have been infecting those animals for millions of years and are now mostly associated with little or no disease [46,47]. There have been at least four strains of HIV-1 identified, that each represents a single transfer from either chimpanzees (M and N strains from the SIVcpz) or from gorillas (O and P strains from SIVgor [48]); the M (major) strain is the pandemic strain and responsible for greater than 98% of human infections [12]. There appear to be many strains of SIV in old world monkeys and the SIVcpz viruses in chimpanzees arose from an ancestral virus that included portions of three different SIVs—from an SIV of an unknown host, an SIV of greater spot-nosed, Mona and mustached monkeys and an SIV from sooty mangabey [12]. Although it is clear that all SIVcpz strains were derived from one common ancestor that contained sequences derived from SIVs from several other species, it is not clear whether that ancestor emerged in chimpanzees, in a previous simian host, or through some combination of co-infections. This virus eventually gave rise to the M and N strains that infected humans, and it also infected gorillas and, in gorillas gave rise to the HIV-1 O and P strains, that were then transmitted to humans. The HIV-1 M strain has caused the global pandemic, while the O strain has caused infection in hundreds of thousands of humans in Central Africa. Other spillovers of lentiviruses from other primates are named HIV-2, and those infect fewer individuals in limited outbreaks. This complex genetic background of HIV shows that the emergence of broadly sustained epidemic or pandemic primate lentiviruses in humans is a difficult process, that only the M and O strains have accomplished.

### (b) Adaptation within intermediate hosts

**Figure 2.**
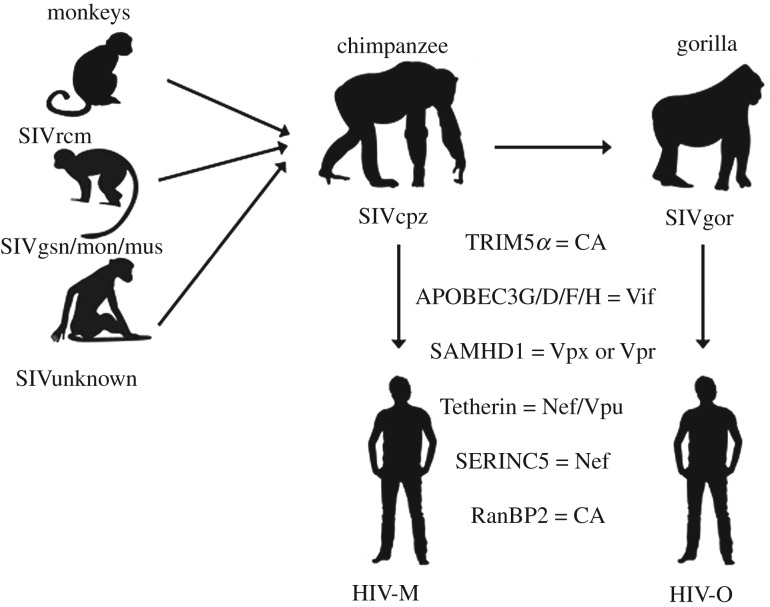
The known host relationships of SIVs and the pathways to the emergence of the two epidemic HIV strains in humans, the M and O strains. The viruses derive from the recombination of viruses infecting three monkey hosts, transfer through chimpanzee for the HIV M strain, and through both the chimpanzee and gorilla for the O strain. The known host barriers and the viral proteins that they interact with are listed; their exact roles and order of contact and evasion are not specifically shown.

The emergence of HIV-1M and O strains each resulted from the transfer to humans of a single virus, which became the ancestor of all the subsequent viruses of that type. The main pathways of emergence in humans and barriers defined are outlined in figure 2. Each strain exemplifies the model where virus spillover from original reservoirs (multiple monkey species) into an intermediate host (chimpanzee for the M strain, in addition to gorilla for the O strain) allowed the virus to gain several changes that allowed it to counteract or become better adapted to several host factors that would otherwise interfere with the virus infection, replication and/or release. The transfer to humans of the M strain was also associated with efficient cell infection, replication and transmission. Similarly, the transfer of the ancestor of the O strain from chimpanzees into gorillas appears to have allowed some additional adaptation, after which the virus transferred to humans. The genetic changes and sequence combinations that allowed successful replication and spread in humans were acquired by complex combinations of recombination, gene rearrangement and acquisition, and point mutations as outlined below. It is assumed that the genetic variation derives from the error- and recombination-prone natures of the viral reverse transcriptase and of the RNA polymerase that produces the viral RNA from the integrated cDNA.

### (c) Interaction with cellular host factors

The emergence of HIV in humans from the original reservoir(s) in monkeys therefore involved two or three host transfers. Each of those steps involved multiple host barriers on the cellular level that the virus needed to overcome before efficient replication and spread were possible. Known host factors that are involved in cell infection and viral adaptation are the cluster of differentiation 4 (CD4) receptor, C–C chemokine receptor type 5 (CCR5) receptor, Tripartite motif-containing protein 5 α form (TRIM5α), apolipoprotein B mRNA editing enzyme, catalytic polypeptide-like (APOBEC) 3D, APOBEC F, APOBEC G, APOBEC 3H, SAM and HD Domain Containing Deoxynucleoside Triphosphate Triphosphohydrolase 1 (SAMHD1), Tetherin, human silencing hub (HUSH) RanBP2 [45]. Each has a degree of host specificity that required one or more modifications or countermeasures from the virus for successful emergence in one or more of the new hosts. The necessary adaptations were achieved through amino acid changes or by the acquisition or modification of viral accessory protein genes: for TRIM5α, this involved mutations in the capsid protein; for APOBECs, it required the presence of the Vif gene; SAMHD1 was countered by the viral Vpr; Tetherin by Nef and/or Vpu; human silencing hub (HUSH) complex by Vpr; RanBP2 interacted with the capsid protein and was countered by capsid mutations. There was also some specificity of attachment or use of the CCR5 protein co-receptor in different hosts [49]. The number of factors viruses had to overcome or become adapted to indicates the complexity of the host-switching process, particularly for the M and O strains. Analysis of the complete SIV and HIV sequences searching for human-specific mutations has shown very few point mutations—indeed, it appears that there is only a single mutation in the gag protein (matrix—MA) associated specifically with adaptation to the human host [50,51].

Each emergent strain, therefore, appears to have bridged the larger host barrier between monkeys and humans by gaining adaptive changes in intermediate hosts, likely allowing the virus to make successful spread among humans possible. However, it is likely that changes in human population demographics and behaviour allowed the spread of the M strain virus from the sites of emergence in central Africa into the rest of the world in the late 1970s and early 1980s.

## 4. Canine parvovirus—emergence in dogs and other hosts

CPV is a member of the Protoparvoviruses, in the Family Parvoviridae. The parvoviruses are small non-enveloped viruses, which form a stable capsid of about 26 nm in diameter, that packages an ssDNA genome of about 5100 bases. The viral DNA replicates through linear double stranded DNA intermediates in the nucleus of the cell using the host cell DNA polymerases, most likely Pol *δ* and some of the accessory polymerases such as the DNA polymerases η and κ produced in response to DNA damage [52].

The example of interest is the emergence of the pandemic CPV in dogs, where the virus apparently spilled over from another host, gained the canine host range and spread worldwide in 1978. The parvovirus properties and many events surrounding the emergence of CPV have also been reviewed recently [53], as well as earlier [27]. The viruses that gave rise to CPV have been known to infect many different hosts within the order Carnivora, and they were first found to cause disease in cats in the 1920s, in raccoons in the 1930s and in mink in the 1940s. Those viruses were variously named feline panleukopenia virus (FPV), raccoon parvovirus or mink enteritis virus, although it has long been recognized that those viruses are related and that viruses from one host could infect some other carnivore hosts. In 1978, new diseases of dogs were recognized which were seen in all regions of the world, with some dogs developing severe gastroenteritis with profuse bloody diarrhoea, while neonatal puppies developed a myocardial disease [54]. The cause was recognized as a parvovirus similar to FPV, which was named CPV type-2 (CPV-2) to distinguish it from the distantly related minute virus of canines, also known as canine bocavirus. Testing of dog sera collected from many parts of the world before 1978 showed positive antibodies in 1974 and 1976 in Europe, but none in other regions of the world until 1978. Sequence analysis showed that CPV was a new virus in dogs, and that all viruses in dogs share a common ancestor present around the mid-1970s, and they were greater than 99% identical to the FPV-like viruses [55,56].

### (a) Receptor binding and the control of host range

**Figure 3.**
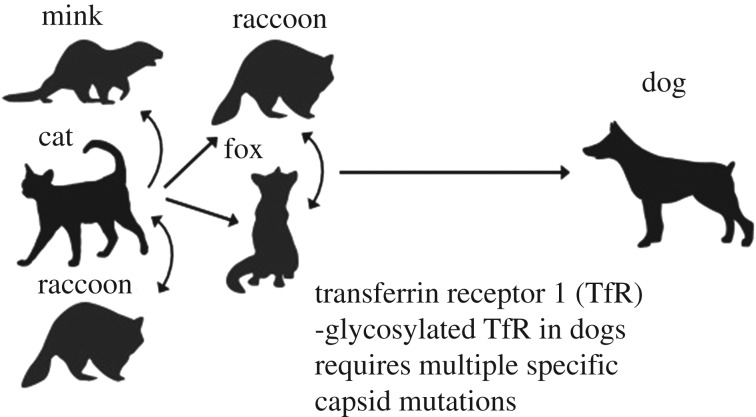
The known host relationships of CPV with the viruses that were long known in other hosts, including cats. The viruses circulate among several carnivore hosts, and cats are likely the major reservoir of FPV. This indicates the possible origin of the ancestor of the CPV in dogs from another host that has not yet been identified.

It is now clear that the canine host range of CPV was primarily associated with changes in the capsid protein gene, with only a few key residues on the capsid surface allowing the virus to infect dog cells [57,58]. The main host range variation and emergence events are outlined in figure 3. CPV uses the cellular transferrin receptor type-1 (TfR) as the receptor for entry [59]. Comparing the sequences of the TfRs from different hosts identified a key change in the canine TfR as an additional glycosylation site in a position where it blocks the binding of FPV-like viruses [57]. That additional glycosylation site is found in a few animal hosts closely related to dogs (wolves, coyotes, possibly golden jackals) [60,61].

CPV is a true pandemic virus, and most of the dogs in the world were infected by the new virus within 1 or 2 years of it becoming widespread [17]. While the CPV-2 strain spread widely in dogs, a variant that contained five additional mutations (named CPV-2a) arose by early 1979 which completely replaced the CPV-2 strain worldwide [53], and that appears to represent a fine tuning of the pandemic virus. All of these viruses cause a systemic infection in animals, infecting through the oro-nasal route, replicating in and spreading between tissues that contain dividing cells, including the lymphoid tissues and the rapidly dividing cells in the crypts of the small intestinal villae [54]. The virus is shed in the faeces of the infected animal, where it is able to survive for long periods allowing efficient transport on fomites. While FPV-like viruses do not bind and infect canine cells in tissue culture, they may replicate in dogs in some tissues after natural exposure, primarily in the thymus [62]. Replication in those internal tissues did not result in efficient shedding of the virus, so those infections are dead-end spillovers. The initial group of capsid changes in CPV-2 gave it the ability to bind the glycosylated canine TfR [59], and allowed infection of the intestinal cells that resulted in efficient shedding and transmission.

### (b) Evolutionary origins of the canine pathogen

In this example, there is frequent exposure of dogs to viruses from other hosts, particularly cats, yet CPV has only arisen once to give a sustained epidemic. It is therefore likely that there are limited combinations of mutations that allow successful use of the canine TfR for efficient cell infection, and that those mutations do not commonly arise in other hosts that produce viruses that expose dogs. The CPV-2 strain that emerged in 1978 did not infect cats, so that host adaptation likely had to occur after infection of dogs—perhaps during the limited replication in the thymus—to allow it to become transmissible in dogs. The emergence of CPV-2a may have involved adaptation of the virus in another host, possibly raccoons [63].

## 5. Common barriers and patterns of virus adaptation to new hosts

Upon analysis of the factors involved in the emergence and onward transmission of the three examples described above, some common patterns become clear. It appears that viruses may encounter one or more barriers in alternative hosts, and will require a number of adaptations in order to achieve sustained transmission in those hosts. Some barriers are outlined below.

### (a) Blockade by mucus and by sialic acids at mucosal surfaces

Many viruses enter the host at mucosal surfaces, and extracellular mucus, therefore, acts as the first barrier to viral infection. Mucus is composed of a variety of different glycans and proteins, which differ widely between various hosts and their tissues. Mucus composition and susceptibility to viral infection also vary depending on the health of the individual, the presence of resident commensal microflora and co-infections, and physiological stressors. Many viruses carry glycosidases, sialidases, or esterases that act as countermeasures against mucin glycans upon entry as well as during egress and shedding of viral particles. Mixed infections of newly transferred viruses with other viruses or bacteria may alter the host mucus and facilitate infection.

Many viruses have glycosylated proteins on their surfaces which are host-specific. Viruses produced in one host will carry an imprinted source glycosylation that is determined by the specific cell or tissue they are produced in. Upon exposure to a new host, these variant glycans (e.g. *N*-glycolyl neuraminic acid (Neu5GC) or α1,3-galactose (α-gal)) may be targeted by preexisting host antibodies to these glycan forms and block the viral infection.

### (b) Receptors

Viral receptors are often key determinants of host and tissues tropism and therefore of host range, and those may be expressed at the cell surface or within the entry pathway. The specificity of virus–receptor interactions may involve host-specific structural interactions that control the affinity of binding, progression to internalization, triggering cell infection, along with key roles for co-receptors where those are present.

### (c) Proteases and protein modification

These control infection and activation of some viruses, and can differ between different hosts and their tissues. These may include the activation of viral glycoproteins by specific cleavage of the protein, as well as recruitment of post-translation modifying pathways such as ubiquitination or phosphorylation, which may be specific for various hosts and tissues and therefore control host susceptibility.

### (d) IFN and other innate intracellular blocks

Innate immune responses, mainly IFN, vary significantly between different animal hosts and often control infection or replication. Viral-encoded countermeasures to IFN or ISGs or their products are often host-specific, and can influence the success of viral transfers. In addition, arboviruses are required to replicate in insect and vertebrate hosts, and therefore need to evade innate immune pathways of both hosts, potentially making vertebrate host shifts difficult. The finding that bats are reservoirs for a number of emerging viruses such as filoviruses and henipaviruses may be in part due to their differential permissive IFN response to viral infection [64].

### (e) Viral transmission processes and population structures

Efficient transmissibility between individuals of the new host is a critical hurdle that must be cleared for an emerging virus to create an epidemic. Processes involved may include the structures and functions of the respiratory secretions that generate aerosols, droplets, or dried particles, materials in faeces and the environment that influence faecal–oral transmission, the nature of the virus-containing materials involved in direct transmission, as well as other viral, host or environmental factors. Persistent infection and shedding provides prolonged opportunities for transmission (e.g. by HIV), while acutely infecting viruses (such as IAV and CPV) are only shed for a few days, so that contact is required (IAV) or survival in faecal matter (CPV). There are many details of transmission that need to be determined, including the physical nature of the shed virus and host components, and the roles of different regions of the respiratory or gastrointestinal tract (or other sites) in facilitating transmission. Invertebrate vectors may impose an addition level of host adaptation, but they may facilitate the spread of a virus in a new host population. All of these potential interactions are still poorly understood—and studying combinations of factors is challenging in experimental animal models of infection and transmission.

The host population structures (of both donor and recipient) play key roles in viral emergence. The density and connectedness of the new host population may determine whether viruses emerge rather than die out. This is particularly true for viruses causing acute infections, which are more prone to burn out before spreading to new patches of host population [65]. Changes in hosts and host populations that alter the likelihood of emergence include population size, density, mobility, behaviour such as sexual practices, and connectedness. Transmission heterogeneity is likely important early in outbreaks of new viruses, when stochastic events will often determine the outcome, and in particular, ‘superspreaders’ may create a large number of secondary infections that allow viruses to overcome demographic barriers [66,67]. Specific control or reduction in those events may be particularly effective in the early control of outbreaks, but that hinges on the identification of potential superspreaders early.

### (f) Virus evolution—general and post-transfer

There are now many thousands of studies of the genetic sequences and variation of viruses, particularly of those that emerge in new hosts. Those confirm that the replication of many viruses is relatively low fidelity and creates many mutations, providing significant amounts of genetic variation in most viral populations. While RNA viruses and retroviruses show frequent mutations and recombination or reassortment, it is now clear that sequence variation is also common in both ssDNA and double-stranded DNA viruses [68]. We are rapidly gaining new insights into the complexities of viral sequences and their structures, as well as the roles of low-level polymorphisms as substrates for rapid evolution [69–71]. As described above, and reviewed elsewhere [72,73], the emergence of new epidemic viruses requires host adaptive mutations, recombination or reassortment, or the acquisition of modifications and accessory genes. Retrospective analysis indicates that those changes occur during the early stages of infection and spread in the new host or within an intermediate host, and that those are required to give increased replication and transmission. It is not generally possible to identify those key mutations before virus emergence, but we can now identify adaptive mutations required for initial epidemic spread, or for additional ‘fine tuning’ of the host range [39]. The functional expression of mutations may be dependent on the sequence context of the virus, creating a significant historical contingency, so that low-risk mutations in one strain may be high risk in even closely related strains.

## 6. Lessons for better understanding and potentially controlling new epidemic viruses

Emerging and re-emerging epidemic viruses are among the greatest threats to the health and wellbeing of humans and other animals (as well as to plants and microbes). The transfer of viruses into new hosts involves the traversing of complex fitness landscapes between old and new hosts and/or invertebrate vectors. Models of emerging epidemic viruses, including those outlined here, show that the process is complex and generally involves multiple steps. Each epidemic examined initiated from a single ancestral virus that made the transition to successful replication and spread in the new host by overcoming a number of barriers, in the form of host-specific proteins or processes that differed between the reservoir and the new outbreak host. Transferred viruses, therefore, had low fitness in their new hosts, and that was improved by the acquisition of multiple mutations, more often in several genes. Intermediate hosts are clearly involved in many examples that have been examined, and those may both give greater exposure to the new outbreak host, and reduce the fitness valley that must be traversed.

### (a) Future approaches to understanding and controlling epidemic emergence

New experimental and computational approaches will further clarify the processes involved. Better analysis of sequence and biological data using the tools of population genetics will also sharpen the conclusions and produce more general insights into the mechanisms of host restriction and how viruses may overcome those. Studying known host range models clarifies the mechanisms that allow specific host entry, replication, transmission and shedding, and reveals the key evolutionary events that have occurred in previous outbreaks. Integrated models of spillover and early transmission can be paired with a knowledge of animal and human viruses. Key challenges include methods to distinguish cases infected directly from animal spillovers (primary cases) and secondary cases resulting from human-to-human transmission, and methods to estimate transmissibility, which are often difficult to obtain at the start of an epidemic. By clarifying the series of steps that are required to go from exposure to spillover to epidemic or pandemic, new models should identify multiple points where strategic interventions could allow us to forestall new viruses before they can emerge to create widespread epidemics.

## Data accessibility

This article has no additional data.

## References

[1] Zhang Y-Z, Wu W-C, Shi M, Holmes EC 2018 The diversity, evolution and origins of vertebrate RNA viruses. Curr. Opin. Virol. 31, 9–16. (10.1016/j.coviro.2018.07.017) 30114593 PMC7102767

[2] Greninger AL 2018 A decade of RNA virus metagenomics is (not) enough. Virus Res. 244, 218–229. (10.1016/j.virusres.2017.10.014) 29055712 PMC7114529

[3] Kumar A, Murthy S, Kapoor A 2017 Evolution of selective-sequencing approaches for virus discovery and virome analysis. Virus Res. 239, 172–179. (10.1016/j.virusres.2017.06.005) 28583442 PMC5819613

[4] Anthony SJ et al. 2013 A strategy to estimate unknown viral diversity in mammals. MBio 4, e00598-13 (10.1128/mBio.00598-13) 24003179 PMC3760253

[5] Plowright RK, Parrish CR, McCallum H, Hudson PJ, Ko AI, Graham AL, Lloyd-Smith JO 2017 Pathways to zoonotic spillover. Nat. Rev. Microbiol. 15, 502–510. (10.1038/nrmicro.2017.45) 28555073 PMC5791534

[6] Viana M, Mancy R, Biek R, Cleaveland S, Cross PC, Lloyd-Smith JO, Haydon DT 2014 Assembling evidence for identifying reservoirs of infection. Trends Ecol. Evol. 29, 270–279. (10.1016/j.tree.2014.03.002) 24726345 PMC4007595

[7] Haydon DT, Cleaveland S, Taylor LH, Laurenson MK 2002 Identifying reservoirs of infection: a conceptual and practical challenge. Emerg. Infect. Dis. 8, 1468–1473. (10.3201/eid0812.010317) 12498665 PMC2738515

[8] Nelson MI, Vincent AL 2015 Reverse zoonosis of influenza to swine: new perspectives on the human–animal interface. Trends Microbiol. 23, 142–153. (10.1016/j.tim.2014.12.002) 25564096 PMC4348213

[9] Palacios G et al 2011 Human metapneumovirus infection in wild mountain gorillas, Rwanda. Emerg. Infect. Dis. 17, 711–713. (10.3201/eid1704.100883) 21470468 PMC3377396

[10] Mena I et al 2016 Origins of the 2009 H1N1 influenza pandemic in swine in Mexico. Elife 5, e16777 (10.7554/eLife.16777) 27350259 PMC4957980

[11] Long JS, Mistry B, Haslam SM, Barclay WS 2018 Host and viral determinants of influenza A virus species specificity. Nat. Rev. Microbiol. 17, 67–81. (10.1038/s41579-018-0115-z) 30487536

[12] Sauter D, Kirchhoff F 2019 Key viral adaptations preceding the AIDS pandemic. Cell Host Microbe 25, 27–38. (10.1016/j.chom.2018.12.002) 30629915

[13] Emanuel J, Marzi A, Feldmann H 2018 Filoviruses: ecology, molecular biology, and evolution. Adv. Virus Res. 100, 189–221. (10.1016/bs.aivir.2017.12.002) 29551136 PMC11056037

[14] Cui J, Li F, Shi Z-L 2018 Origin and evolution of pathogenic coronaviruses. Nat. Rev. Microbiol. 17, 181–192. (10.1038/s41579-018-0118-9) PMC7097006 30531947

[15] Wang L-F, Anderson DE 2019 Viruses in bats and potential spillover to animals and humans. Curr. Opin. Virol. 34, 79–89. (10.1016/j.coviro.2018.12.007) 30665189 PMC7102861

[16] Thibault PA, Watkinson RE, Moreira-Soto A, Drexler JF, Lee B 2017 Zoonotic potential of emerging paramyxoviruses: knowns and unknowns. Adv. Virus Res. 98, 1–55. (10.1016/bs.aivir.2016.12.001) 28433050 PMC5894875

[17] Hoelzer K, Parrish CR 2010 The emergence of parvoviruses of carnivores. Vet. Res. 41, 39 (10.1051/vetres/2010011) 20152105 PMC2844231

[18] Gutiérrez-Bugallo G, Piedra LA, Rodriguez M, Bisset JA, Lourenço-de-Oliveira R, Weaver SC, Vasilakis N, Vega-Rúa A 2019 Vector-borne transmission and evolution of Zika virus. Nat. Ecol. Evol. 3, 561–569. (10.1038/s41559-019-0836-z) 30886369 PMC8900209

[19] Humphreys M 2018 The influenza of 1918: evolutionary perspectives in a historical context. Evol. Med. Public Health 2018, 219–229. (10.1093/emph/eoy024) 30410762 PMC6218637

[20] Barclay W, Openshaw P 2018 The 1918 influenza pandemic: one hundred years of progress, but where now? Lancet Respir. Med. 6, 588–589. (10.1016/S2213-2600(18)30272-8) 29941354

[21] Yoon S-W, Webby RJ, Webster RG 2014 Evolution and ecology of influenza A viruses. Curr. Top. Microbiol. Immunol. 385, 359–375. (10.1007/82_2014_396) 24990620

[22] Vincent A et al 2014 Review of influenza A virus in swine worldwide: a call for increased surveillance and research. Zoonoses Public Health 61, 4–17. (10.1111/zph.12049) 23556412

[23] Hayward JJ, Dubovi EJ, Scarlett JM, Janeczko S, Holmes EC, Parrish CR 2010 Microevolution of canine influenza virus in shelters and its molecular epidemiology in the United States. J. Virol. 84, 12 636–12 645. (10.1128/JVI.01350-10) PMC3004329 20943966

[24] Puryear WB et al 2016 Prevalence of influenza A virus in live-captured North Atlantic gray seals: a possible wild reservoir. Emerg. Microbes Infect. 5, e81 (10.1038/emi.2016.77) 27485496 PMC5034098

[25] He W et al 2018 Emergence and adaptation of H3N2 canine influenza virus from avian influenza virus: an overlooked role of dogs in interspecies transmission. Transbound. Emerg. Dis. 66, 842–851. (10.1111/tbed.13093) 30520554

[26] Ciminski K, Thamamongood T, Zimmer G, Schwemmle M 2017 Novel insights into bat influenza A viruses. J. Gen. Virol. 98, 2393–2400. (10.1099/jgv.0.000927) 28906230 PMC5725989

[27] Parrish CR, Kawaoka Y 2005 The origins of new pandemic viruses: the acquisition of new host ranges by canine parvovirus and influenza A viruses. Annu. Rev. Microbiol. 59, 553–586. (10.1146/annurev.micro.59.030804.121059) 16153179

[28] Christman MC, Kedwaii A, Xu J, Donis RO, Lu G 2011 Pandemic (H1N1) 2009 virus revisited: an evolutionary retrospective. Infect. Genet. Evol. 11, 803–811. (10.1016/j.meegid.2011.02.021) 21382522 PMC3141221

[29] Lipsitch M et al 2016 Viral factors in influenza pandemic risk assessment. Elife 5, e18491 (10.7554/eLife.18491) 27834632 PMC5156527

[30] Wilks S, de Graaf M, Smith DJ, Burke DF. 2012 A review of influenza haemagglutinin receptor binding as it relates to pandemic properties. Vaccine 30, 4369–4376. (10.1016/j.vaccine.2012.02.076) 22682293 PMC3372863

[31] de Graaf M, Fouchier RAM 2014 Role of receptor binding specificity in influenza A virus transmission and pathogenesis. EMBO J. 33, 823–841. (10.1002/embj.201387442) 24668228 PMC4194109

[32] Peng W et al 2017 Recent H3N2 viruses have volved specificity for extended, branched human-type receptors, conferring potential for increased avidity. Cell Host Microbe 21, 23–34. (10.1016/j.chom.2016.11.004) 28017661 PMC5233592

[33] Peng W, Bouwman KM, McBride R, Grant OC, Woods RJ, Verheije MH, Paulson JC, de Vries RP. 2018 Enhanced human-type receptor binding by ferret transmissible H5N1 with a K193T mutation. J. Virol. 92, e02016-17 (10.1128/JVI.02016-17) 29491160 PMC5923085

[34] Boivin S, Hart DJ 2011 Interaction of the influenza A virus polymerase PB2 C-terminal region with importin alpha isoforms provides insights into host adaptation and polymerase assembly. J. Biol. Chem. 286, 10 439–10 448. (10.1074/jbc.M110.182964) PMC3060497 21216958

[35] Long JS et al 2016 Species difference in ANP32A underlies influenza A virus polymerase host restriction. Nature 529, 101–104. (10.1038/nature16474) 26738596 PMC4710677

[36] Lakdawala SS, Fodor E, Subbarao K 2016 Moving on out: transport and packaging of influenza viral RNA into virions. Annu. Rev. Virol. 3, 411–427. (10.1146/annurev-virology-110615-042345) 27741407

[37] Shim JM, Kim J, Tenson T, Min J-Y, Kainov DE 2017 Influenza virus infection, interferon response, viral counter-response, and apoptosis. Viruses 9, 223 (10.3390/v9080223) PMC5580480 28805681

[38] Nogales A, Martinez-Sobrido L, Topham DJ, DeDiego ML 2018 Modulation of innate immune responses by the influenza A NS1 and PA-X proteins. Viruses 10, 708 (10.3390/v10120708) PMC6315843 30545063

[39] Pepin KM, Lass S, Pulliam JRC, Read AF, Lloyd-Smith JO 2010 Identifying genetic markers of adaptation for surveillance of viral host jumps. Nat. Rev. Microbiol. 8, 802–813. (10.1038/nrmicro2440) 20938453 PMC7097030

[40] Chauché C et al 2018 Mammalian adaptation of an avian influenza A virus involves stepwise changes in NS1. J. Virol. 92, e01875-17 (10.1128/JVI.01875-17) 29237841 PMC5809720

[41] Russell CJ, Hu M, Okda FA 2018 Influenza hemagglutinin protein stability, activation, and pandemic risk. Trends Microbiol. 26, 841–853. (10.1016/j.tim.2018.03.005) 29681430 PMC6150828

[42] Wang W, DeFeo CJ, Alvarado-Facundo E, Vassell R, Weiss CD 2015 Intermonomer interactions in hemagglutinin subunits HA1 and HA2 affecting hemagglutinin stability and influenza virus infectivity. J. Virol. 89, 10 602–10 611. (10.1128/JVI.00939-15) PMC4580181 26269180

[43] Holmes KK, Bertozzi S, Bloom BR, Jha P, Gelband H, DeMaria LM, Horton S 2017 Major infectious diseases: key messages from disease control priorities, third edition. In Major infectious diseases (eds Holmes KK, Bertozzi S, Bloom BR, Jha P), Washington, DC: The International Bank for Reconstruction and Development/The World Bank. 30212102

[44] Simon V, Bloch N, Landau NR 2015 Intrinsic host restrictions to HIV-1 and mechanisms of viral escape. Nat. Immunol. 16, 546–553. (10.1038/ni.3156) 25988886 PMC6908429

[45] Kluge SF, Sauter D, Kirchhoff F 2015 SnapShot: antiviral restriction factors. Cell 163, 774–774.e1. (10.1016/j.cell.2015.10.019) 26496613

[46] McCarthy KR, Kirmaier A, Autissier P, Johnson WE 2015 Evolutionary and functional analysis of old world primate TRIM5 reveals the ancient emergence of primate lentiviruses and convergent evolution targeting a conserved capsid interface. PLoS Pathog. 11, e1005085 (10.1371/journal.ppat.1005085) 26291613 PMC4546234

[47] Gilbert C, Maxfield DG, Goodman SM, Feschotte C 2009 Parallel germline infiltration of a lentivirus in two Malagasy lemurs. PLoS Genet. 5, e1000425 (10.1371/journal.pgen.1000425) 19300488 PMC2651035

[48] D'arc M et al 2015 Origin of the HIV-1 group O epidemic in western lowland gorillas. Proc. Natl Acad. Sci. USA 112, E1343–E1352. (10.1073/pnas.1502022112) 25733890 PMC4371950

[49] Wetzel KS, Elliott STC, Collman RG 2018 SIV coreceptor specificity in natural and non-natural host infection: implications for cell targeting and differential outcomes from infection. Curr. HIV Res. 16, 41–51. (10.2174/1570162X15666171124121805) 29173179

[50] Wain LV et al 2007 Adaptation of HIV-1 to its human host. Mol. Biol. Evol. 24, 1853–1860. (10.1093/molbev/msm110) 17545188 PMC4053193

[51] Bibollet-Ruche F et al 2012 Efficient SIVcpz replication in human lymphoid tissue requires viral matrix protein adaptation. J. Clin. Invest. 122, 1644–1652. (10.1172/JCI61429) 22505456 PMC3336991

[52] Deng X, Xu P, Zou W, Shen W, Peng J, Liu K, Engelhardt JF, Yan Z, Qiu J 2017 DNA damage signaling is required for replication of human bocavirus 1 DNA in dividing HEK293 cells. J. Virol. 91, e01831-16 (10.1128/JVI.01831-16) 27733644 PMC5165215

[53] Kailasan S, Agbandje-McKenna M, Parrish CR 2015 Parvovirus family conundrum: what makes a killer? Annu. Rev. Virol. 2, 425–450. (10.1146/annurev-virology-100114-055150) 26958923

[54] Parrish CR 1995 Pathogenesis of feline panleukopenia virus and canine parvovirus. Baillieres Clin. Haematol. 8, 57–71. (10.1016/S0950-3536(05)80232-X) 7663051 PMC7134857

[55] Hoelzer K, Shackelton LA, Parrish CR, Holmes EC 2008 Phylogenetic analysis reveals the emergence, evolution and dispersal of carnivore parvoviruses. J. Gen. Virol. 89, 2280–2289. (10.1099/vir.0.2008/002055-0) 18753238 PMC2735869

[56] Allison AB, Kohler DJ, Fox KA, Brown JD, Gerhold RW, Shearn-Bochsler VI, Dubovi EJ, Parrish CR, Holmes EC 2013 Frequent cross-species transmission of parvoviruses among diverse carnivore hosts. J. Virol. 87, 2342–2347. (10.1128/JVI.02428-12) 23221559 PMC3571474

[57] Palermo LM, Hueffer K, Parrish CR 2003 Residues in the apical domain of the feline and canine transferrin receptors control host-specific binding and cell infection of canine and feline parvoviruses. J. Virol. 77, 8915–8923. (10.1128/JVI.77.16.8915-8923.2003) 12885908 PMC167234

[58] Callaway HM, Feng KH, Lee DW, Allison AB, Pinard M, McKenna R, Agbandje-McKenna M, Hafenstein S, Parrish CR 2017 Parvovirus capsid structures required for infection: mutations controlling receptor recognition and protease cleavages. J. Virol. 91, e01871-16 (10.1128/JVI.01871-16) 27847360 PMC5215354

[59] Callaway HM, Welsch K, Weichert W, Allison AB, Hafenstein SL, Huang K, Iketani S, Parrish CR 2018 Complex and dynamic interactions between parvovirus capsids, transferrin receptors and antibodies control cell infection and host range. J. Virol. 92, e00460-18 (10.1128/JVI.00460-18) 29695427 PMC6002733

[60] Kaelber JT, Demogines A, Harbison CE, Allison AB, Goodman LB, Ortega AN, Sawyer SL, Parrish CR 2012 Evolutionary reconstructions of the transferrin receptor of Caniforms supports canine parvovirus being a re-emerged and not a novel pathogen in dogs. PLoS Pathog. 8, e1002666 (10.1371/journal.ppat.1002666) 22570610 PMC3342950

[61] Allison AB, Kohler DJ, Ortega A, Hoover EA, Grove DM, Holmes EC, Parrish CR 2014 Host-specific parvovirus evolution in nature is recapitulated by *in vitro* adaptation to different carnivore species. PLoS Pathog. 10, e1004475 (10.1371/journal.ppat.1004475) 25375184 PMC4223063

[62] Truyen U, Evermann JF, Vieler E, Parrish CR 1996 Evolution of canine parvovirus involved loss and gain of feline host range. Virology 215, 186–189. (10.1006/viro.1996.0021) 8560765

[63] Allison AB et al 2012 Role of multiple hosts in the cross-species transmission and emergence of a pandemic parvovirus. J. Virol. 86, 865–872. (10.1128/JVI.06187-11) 22072763 PMC3255849

[64] Kuzmin IV, Schwarz TM, Ilinykh PA, Jordan I, Ksiazek TG, Sachidanandam R, Basler CF, Bukreyev A 2017 Innate immune responses of bat and human cells to filoviruses: commonalities and distinctions. J. Virol. 91, e02471-16 (10.1128/JVI.02471-16) 28122983 PMC5375674

[65] Cross PC, Lloyd-Smith JO, Johnson PLF, Getz WM 2005 Duelling timescales of host movement and disease recovery determine invasion of disease in structured populations. Ecol. Lett. 8, 587–595. (10.1111/j.1461-0248.2005.00760.x)

[66] Lloyd-Smith JO, Schreiber SJ, Kopp PE, Getz WM 2005 Superspreading and the effect of individual variation on disease emergence. Nature 438, 355–359. (10.1038/nature04153) 16292310 PMC7094981

[67] Wong G, Liu W, Liu Y, Zhou B, Bi Y, Gao GF 2015 MERS, SARS, and Ebola: the role of super-spreaders in infectious disease. Cell Host Microbe 18, 398–401. (10.1016/j.chom.2015.09.013) 26468744 PMC7128246

[68] Peck KM, Lauring AS 2018 Complexities of viral mutation rates. J. Virol. 92, e01031-17 (10.1128/JVI.01031-17) 29720522 PMC6026756

[69] Lyons DM, Lauring AS 2018 Mutation and epistasis in influenza virus evolution. Viruses 10, 407 (10.3390/v10080407) PMC6115771 30081492

[70] Poirier EZ, Vignuzzi M 2017 Virus population dynamics during infection. Curr. Opin. Virol. 23, 82–87. (10.1016/j.coviro.2017.03.013) 28456056

[71] Dolan PT, Whitfield ZJ, Andino R 2018 Mechanisms and concepts in RNA virus population dynamics and evolution. Annu. Rev. Virol. 5, 69–92. (10.1146/annurev-virology-101416-041718) 30048219 PMC13283306

[72] Dennehy JJ 2017 Evolutionary ecology of virus emergence. Ann. N. Y. Acad. Sci. 1389, 124–146. (10.1111/nyas.13304) 28036113 PMC7167663

[73] Parrish CR, Holmes EC, Morens DM, Park E-C, Burke DS, Calisher CH, Laughlin CA, Saif LJ, Daszak P 2008 Cross-species virus transmission and the emergence of new epidemic diseases. Microbiol. Mol. Biol. Rev. 72, 457–470. (10.1128/MMBR.00004-08) 18772285 PMC2546865

